# Evolving concepts of liver fibrogenesis provide new diagnostic and therapeutic options

**DOI:** 10.1186/1476-5926-6-7

**Published:** 2007-07-30

**Authors:** Olav A Gressner, Ralf Weiskirchen, Axel M Gressner

**Affiliations:** 1 Institute of Clinical Chemistry and Pathobiochemistry, RWTH-University Hospital, Aachen, Germany

## Abstract

Despite intensive studies, the clinical opportunities for patients with fibrosing liver diseases have not improved. This will be changed by increasing knowledge of new pathogenetic mechanisms, which complement the "canonical principle" of fibrogenesis. The latter is based on the activation of hepatic stellate cells and their transdifferentiation to myofibroblasts induced by hepatocellular injury and consecutive inflammatory mediators such as TGF-β. Stellate cells express a broad spectrum of matrix components. New mechanisms indicate that the heterogeneous pool of (myo-)fibroblasts can be supplemented by epithelial-mesenchymal transition (EMT) from cholangiocytes and potentially also from hepatocytes to fibroblasts, by influx of bone marrow-derived fibrocytes in the damaged liver tissue and by differentiation of a subgroup of monocytes to fibroblasts after homing in the damaged tissue. These processes are regulated by the cytokines TGF-β and BMP-7, chemokines, colony-stimulating factors, metalloproteinases and numerous trapping proteins. They offer innovative diagnostic and therapeutic options. As an example, modulation of TGF-β/BMP-7 ratio changes the rate of EMT, and so the simultaneous determination of these parameters and of connective tissue growth factor (CTGF) in serum might provide information on fibrogenic activity. The extension of pathogenetic concepts of fibrosis will provide new therapeutic possibilities of interference with the fibrogenic mechanism in liver and other organs.

## Introduction

Experimental and clinical studies of the past twenty years or so provide a detailed knowledge of structure and composition of extracellular matrix (ECM) in normal and fibrotic liver tissue [1,2], of the cellular origin of the various matrix components [3], of the cytokine- and growth factor-regulated stimulation of ECM synthesis (fibrogenesis) and regulation of matrix degradation (fibrolysis) [4-6], of several genetic conditions predisposing for fibrogenesis [7,8], and of multiple, experimentally successful therapeutic approaches [9]. However, up to now the clinical benefit derived from basic research in the context of translational medicine is scarce with regard to an effective, harmless and site-directed antifibrotic therapy and approved non-invasive diagnostic measures of the activity of fibrogenesis ("grading") and/or of the extent of the fibrotic organ transition ("staging") using serum parameters [10]. The failure of clinical success boosts current research on fibrosis and fibrogenesis not only of the liver, but also of the lung, kidney, pancreas, heart, skin, bone marrow, and other organs with the result that during the last four to five years very important new insights into the pathogenesis of fibrosis and of related diagnostic and therapeutic options have been made [11]. Evolving pathogenetic concepts supplement the so called "canonical principle" of liver fibrogenesis, which has been worked out in detail during the last twenty years and which is based, in principle, on the activation of hepatic stellate cells (HSC).

### The "canonical principle" of liver fibrogenesis

**Figure 1:**
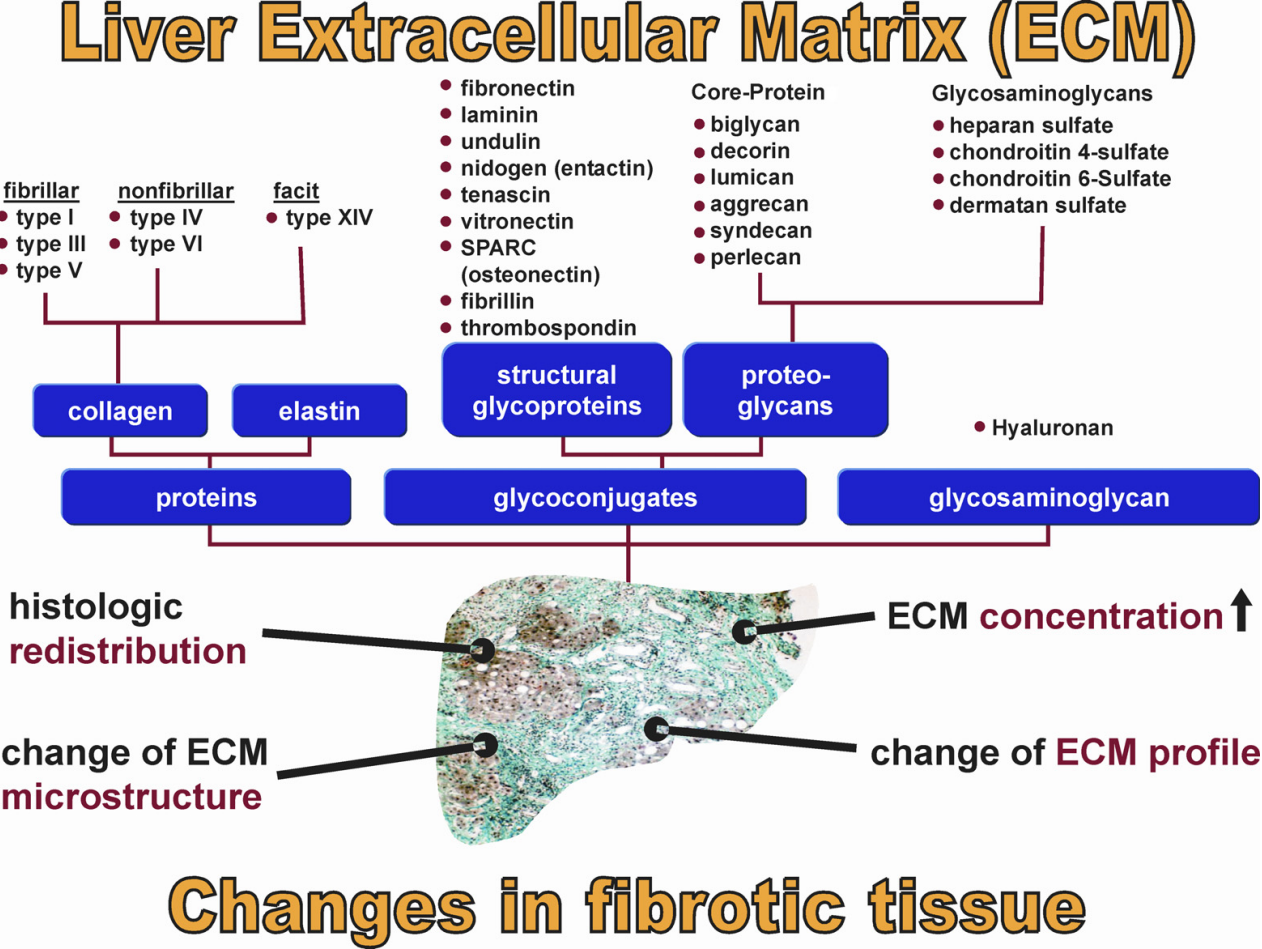
**Matrix elements and fibrotic changes**. Major components of the extracellular matrix (connective tissue) of the liver and the four most important changes in the fibrotic matrix.

**Figure 2:**
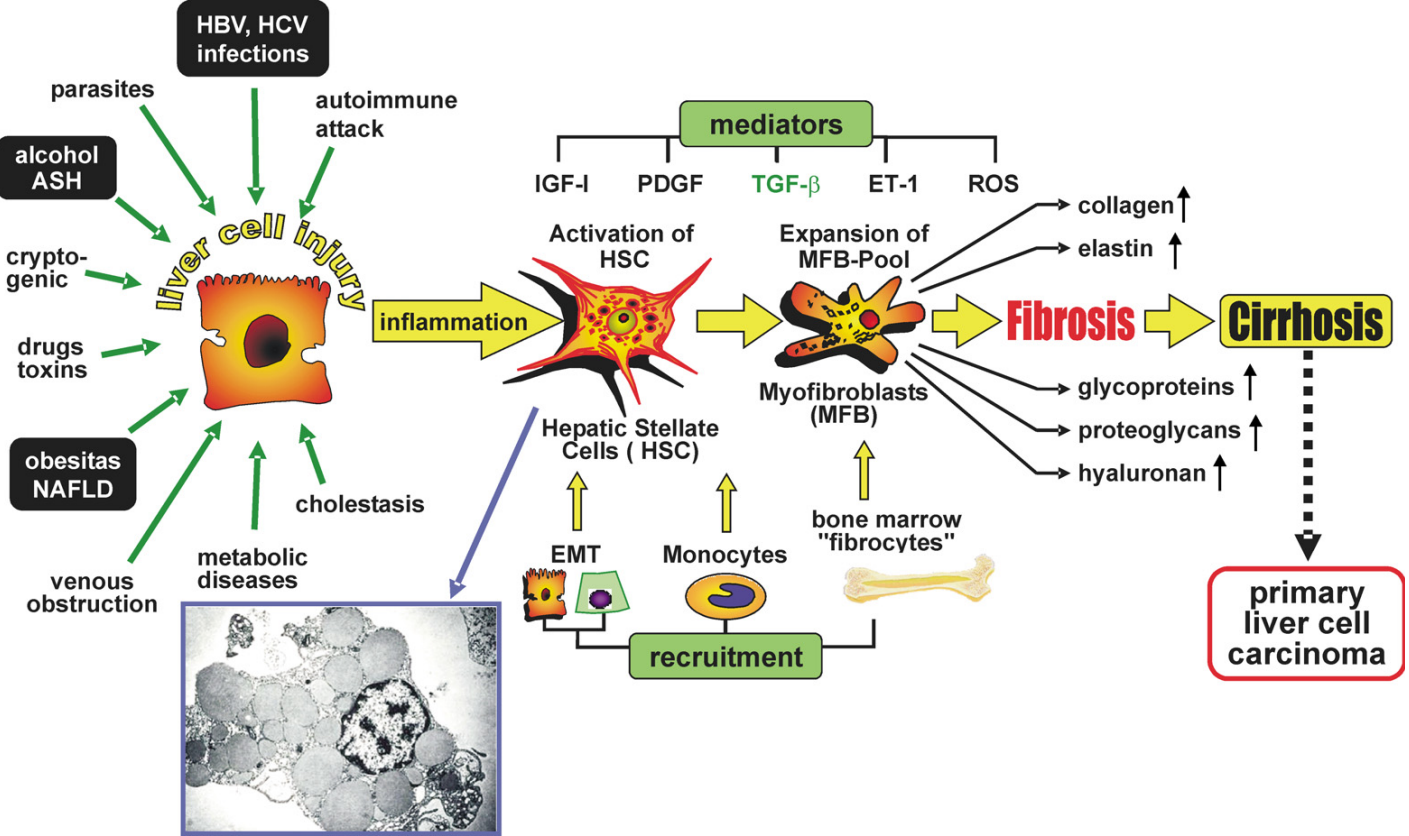
**Formal pathogenesis of liver fibrosis (fibrogenesis)**. The "canonical principle" of fibrogenesis starts with necrosis or apoptosis of hepatocytes and inflammation-connected activation of hepatic stellate cells (HSC triggering), their transdifferentiation to myofibroblasts with enhanced expression and secretion of extracellular matrix and matrix deposition (fibrosis). The latter is a precondition for cirrhosis. New pathogenetic mechanisms concern the influx of bone marrow-derived cells (fibrocytes) and of circulating monocytes and their TGF-β driven differentiation to fibroblasts in the damaged liver tissue. A further new mechanism is epithelial-mesenchymal transition (EMT) of bile duct epithelial cells and potentially of hepatocytes. All three complementary mechanisms enlarge the pool of matrix-synthesizing (myo-)fibroblasts in the damaged liver. The most important fibrogenic mediators are transforming growth factor (TGF)-β, platelet-derived growth factor (PDGF), insulin-like growth factor 1 (IGF-1), endothelin-1 (ET-1), and reactive oxygen species (ROS including hydroxyl radicals, superoxid anions). Abbreviations: ASH – alcoholic steatohepatitis; NAFLD – non-alcoholic fatty liver disease. Inset shows an electron micrograph of HSC with numerous lipid droplets indenting the nucleus.

**Figure 3:**
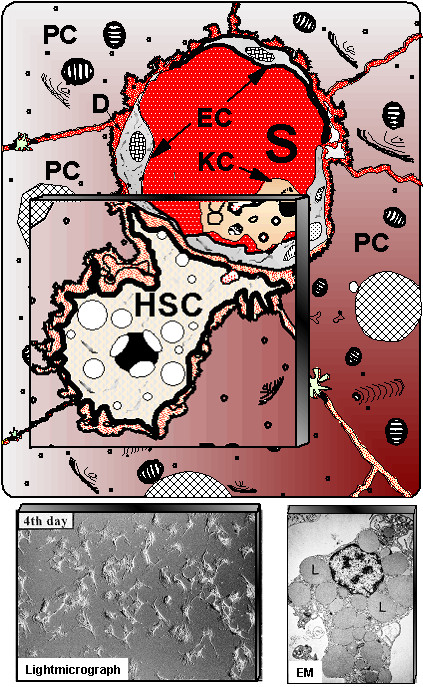
**Schematic presentation of hepatic stellate cells (HSC) located in the vicinity of adjacent hepatocytes (PC) beneath the sinusoidal endothelial cells (EC)**. S – liver sinusoids; KC – Kupffer cells. Down left shows cultured HSC at light-microscopy, whereas at down right electron microscopy (EM) illustrates numerous fat vacuoles (L) in a HSC, in which retinoids are stored.

**Figure 4:**
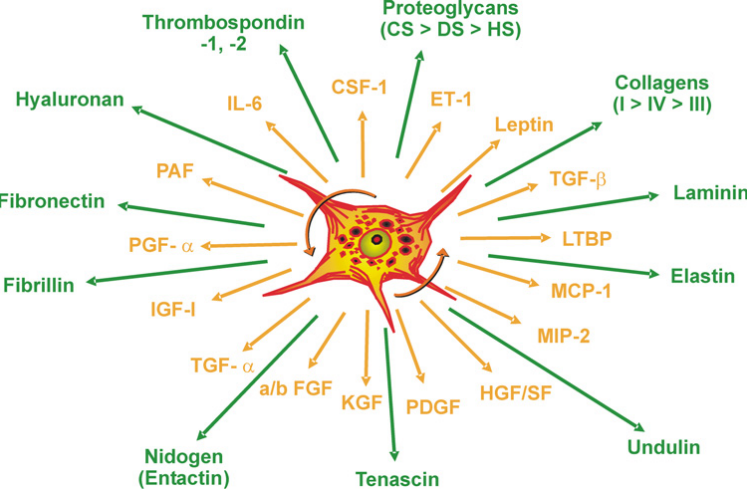
**Compilation of the most important components of extracellular matrix and of mediators synthesized by activated hepatic stellate cells (HSC)**. Abbreviations: CF – colony-stimulating factor; ET – endothelin; HGF – hepatocyte growth factor; IGF – insulin-like growth factor; KGF – keratinocyte growth factor; LTBP – latent TGF-β binding protein; MCP – monocyte chemotactic peptide; MIP – macrophage inflammatory protein; PAF – platelet activating factor; PDGF – platelet-derived growth factor; PGF – prostaglandin F; SF – scatter factor; TGF – transforming growth factor.

**Figure 5:**
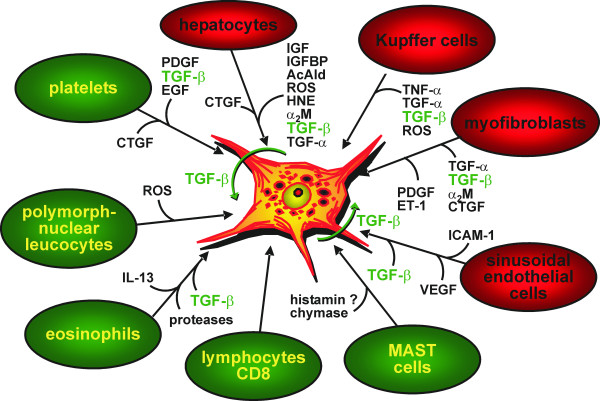
**Cellular interactions**. Synopsis of cellular interactions of resident liver cells (red) and immigrated inflammatory cells (green) with hepatic stellate cells in the process of activation and transdifferentiation to myofibroblasts. The most important paracrine mediators are given.

**Figure 6:**
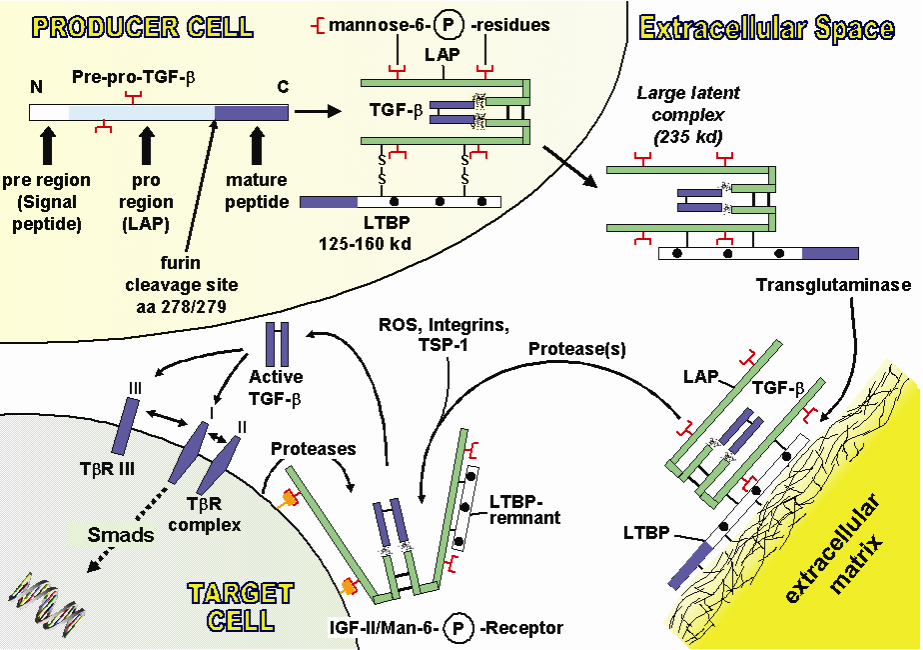
**Extracellular matrix and TGF-β**. Schematic presentation of intracellular TGF-β synthesis, secretion and extracellular immobilization via transglutaminase-dependent fixation of the large latent TGF-β binding protein (LTBP) to extracellular matrix, release by proteases and activation of the latent TGF-β complex by reactive oxygen species (ROS), specific integrins, thrombospondin-1 (TSP-1) or proteases with release of the active TGF-β homodimer, which binds to TGF-β receptors (TβR) III, II, and I to initiate the intracellular signalling cascade of Smad phosphorylation. Regulation of TGF-β occurs at the transcriptional level and, most importantly, by extracellular activation. LAP – latency associated peptide.

Fibrosis is characterized by a severalfold increase of the extracellular matrix that comprises collagens, structural glycoproteins, sulphated proteoglycans and hyaluronan, by a histological redistribution with preferred initial matrix deposition in the subendothelial space of Disse leading to the formation of an incomplete subendothelial basement membrane creating additional diffusion barriers between hepatocytes and the liver sinusoid ("capillarization of sinusoids"), and by changes in the microstructure of collagens (*e.g*., degree of hydroxylation of prolin and lysin), glycoproteins (variations of the carbohydrate structure) and proteoglycans (changes of the degree of sulfation of the glycosaminoglycan side chains) (Fig. 1). It is known for a long time that the increase of ECM in the parenchyma is not a passive process caused by condensation of pre-existing septa of connective tissue due to necrotic and apoptotic collapse of the parenchyma, instead, it is an active biosynthetic process, which is attributed to stimulated matrix production in portal or peribiliary fibroblasts and, in particular, in contractile myofibroblasts (MFB) localized initially in the subendothelial space of Disse. The development of MFB is the result of a multi-step sequence, which originates from liver cell necrosis induced by various noxious agents (toxic, immunologic) [12,13] (Fig. 2). As a consequence, HSC, formerly called vitamin A-storing cells, fat-storing cells, arachnocytes, and Ito-cells [14,15], and localized in the immediate vicinity of hepatocytes are activated (Fig. 3). HSC are liver pericytes, which embrace with thorn-like microprojections the endothelial cell layer of the sinusoids providing physical contact not only to sinusoidal endothelial cells, but also with the cell body to the hepatocytes [16]. HSC constitute about 1/3 of the non-parenchymal cell population (Kupffer cells, endothelial cells, HSC) and about 15% of total liver resident cells including hepatocytes. The "hepatic stellate cell index", *i. e*., the number of HSCs per 1000 hepatocytes was estimated to be 109 in the healthy rat liver [17]. The spindle-like cell body of HSC contains multiple triglyceride-rich vacuoles, in which vitamin A metabolites (retinoids) are dissolved and stored [18]. About 85% of the vitamin A of the liver is found in HSC. Additional functions of these cells were recently discovered: they seem to play a role as antigen presenting cells (APC) [19-21], as CD133^+ ^progenitor-cells with the ability to differentiate to progenitor endothelial cells and hepatocytes suggesting important roles in liver regeneration and repair [22], they are involved in endocytosis of apoptotic parenchymal cells [23,24], in secretion of apolipoproteins, matrix metalloproteinases (MMPs), respective MMP-inhibitors (TIMPs) [25,26] and growth factors [3], in the support of liver regeneration through promotion of hepatocyte proliferation involving the neurotrophin receptor p75 [27], in regulation of angiogenesis and vascular remodelling through secretion of angiogenic factors [28], and in hemodynamic functions since activated HSC contract under stimulation by thromboxan, prostaglandin F2, angiotensin II, vasopressin, and endothelin-1 leading to sinusoidal constriction [29-32]. Some of these functions, however, are not expressed in the quiescent status of HSC, but are symptoms of their activation triggered by inflammatory mediators in consequence of liver cell damage. The activation of HSC leads to the expression of α-smooth-muscle actin and a loss of fat vacuoles combined with a decrease of retinoids, but increases their contractility and strongly their capacity to express and secrete a broad spectrum of matrix components [3]. The activation process includes proliferation and phenotypic transdifferentiation of HSC to MFB, but both processes are not causally related. In the "canonical principle" of fibrogenesis HSC-derived MFB have the core competency not only for matrix synthesis, but also for the expression and secretion of numerous pro- and anti-inflammatory cytokines and growth factors (Fig. 4). They have a highly synthetic phenotype characterized by a hypertrophic rough endoplasmic reticulum containing ribosomes necessary for the synthesis of export proteins. The mechanism of fibrogenic activation and transdifferentiation of HSC to MFB can be summarized in a three-step cascade model [33], which is initialized by the pre-inflammatory phase due to direct paracrine activation of HSC by necrotic (apoptotic?) hepatocytes with release of activating cytokines supplemented by a loss of mito-inhibitory cell surface heparan sulfate [34-38]. The growth promoting activity of hepatocytes, partially due to IGF-1 and respective IGF-binding proteins [13], is released from damaged cells and parallels the elevation of lactate dehydrogenase and aspartate aminotransferase as known leakage enzymes of hepatocytes [39]. In the following inflammatory phase, the pre-activated HSC are further stimulated in a paracrine mode by invaded leucocytes and thrombocytes [40], but also by activated Kupffer cells [36,41-44], sinusoidal endothelial cells and hepatocytes [13,34,37] to transdifferentiate to MFB. The consecutive postinflammatory phase is characterized by the secretion of stimulating cytokines from MFB and interacting matrix components. Some of these cytokines can stimulate in an autocrine way MFB and in a paracrine mode resting HSC. Thus, the postinflammatory phase contributes significantly to the perpetuation of the fibrogenic process, even after elimination or reduction of the pre-inflammatory and inflammatory phases. Activation and transdifferentiation of HSC is the result of extensive interactions with liver-resident and non-resident cells (Fig. 5). Most relevant cellular mediators are reactive oxygen species (hydroxyl radicals, oxygen radicals, superoxide anions, hydrogen peroxide) produced by activated Kupffer cells [41,45], the stimulated NAD(P)H oxidase activity of HSC [46] phagocytosing apoptotic bodies [24], the cytochrome P4502E1 (CYP2E1) pathway of ethanol-metabolizing hepatocytes [47], and leucocytes [48]. In addition, acetaldehyde of ethanol-exposed hepatocytes [49-52] and tissue hypoxia [53] promote the activation of HSC. Among the peptide mediators transforming growth factor (TGF)-β turned out to be the fibrogenic master cytokine [54-56]. Additional cytokines and growth factors involved in fibrogenesis are platelet-derived growth factor B and D (PDGF-B and PDGF-D), endothelin-1, several fibroblast growth factors (FGFs), insulin-like growth factor I, tumor necrosis factor (TNF)-α, adipocytokines (leptin, adiponectin), and others, which are partly bound as "crinopectins" [57] to the extracellular matrix [58]. The matrix serves as a sponge for several of these growth factors fixed in a covalent or non-covalent manner to fibronectin, proteoglycans and collagens. TGF-β, which is secreted in a high molecular (large) latent form (Fig. 6) by HSC/MFB, sinusoidal endothelial cells, and Kupffer cells and released by destructed thrombocytes and hepatocytes [59,60] initiates not only the activation of HSC to MFB, but also enhances matrix gene expression, decreases their degradation by down-modulation of matrix metalloproteinases and up-regulation of specific inhibitors (tissue inhibitors of metalloproteinases, TIMPs), induces apoptosis of hepatocytes [61,62], and inhibits (together with activin A) liver cell proliferation [63,64]. Extracellular activation of latent TGF-β by proteases, oxygen radicals, thrombospondin type I, and α_v_β_1_, α_1_β_6 _integrins is an important step in the regulation of TGF-β bioavailability [65]. Antagonism of TGF-β [66] or inhibition of its intracellular Smad-signaling cascade by specific inhibitors [67] leads to a significant retardation of HSC activation and thus to a sustained antifibrotic effect. Interestingly, TGF-β response and signalling are modulated during transdifferentiation of HSC to MFB leading to their partial TGF-β insensitivity [68]. This observation suggests a role of TGF-β in the initiation of HSC activation *in vivo *but not a TGF-β requirement for the entire transdifferentiation process [69]. The activation of HSC to MFB in the chronically inflamed liver is partially mimicked by primary cultures of HSC, if these cells are plated on plastic surfaces instead of extracellular matrices with no possibility of integrin anchorage [70]. The model was previously suggested as a valuable tool for studying the role of HSC in chronic liver disease [71]. Accordingly, this cell culture system is quite extensively used for testing of potentially antifibrotic drugs, *e.g*., PPAR-γ agonists [72], trichostatin A, pirfenidone, halofuginone, scavengers of reactive oxygen species (α-tocopherol, resveratrol, quercetin, curcumine), protease inhibitors, and others. However, a comparison of the gene expression profiles of HSC activated *in vivo *by bile-duct ligation or CCl_4_-injury with that of culture activated HSC could establish major differences [73]. Thus, culture activation does not properly reflect genetic reprogramming of disease-driven HSC activation. Factors in the microenvironment such as Kupffer cells and lipopolysaccharides were identified to be relevant for the observed differences [73]. Due to morphological and functional intralobular (zonal) heterogeneity of HSC [74-76], the processes of activation and transdifferentiation *in situ *are slightly different, which is also dependent on the different zonal vulnerability of hepatocytes. Accordingly, periveneous hepatocytes around the central vein (acinus zone 3) are the most sensitive and fibrogenesis, *e. g*., in alcoholic liver injury, starts here first [77]. The heterogeneity of HSC or MFB is not confined to their topographic localization, but can also result from their origin, in particular since morphological and functional criteria and the response to growth factors point to different sources of origin of MFB [78]. As an example, HSC express the cytoskeleton proteins glial fibrillary acidic protein and desmin, which are absent in MFB and the matrix protein reelin. MFB, however, almost exclusively synthesize the matrix protein fibulin [79,80]. Using a dual reporter gene transgenic mouse model of secondary biliary fibrosis (bile duct ligation) it could be shown that peribiliary, parenchymal and vascular fibrogenic cells expressed both transgenes (α-smooth muscle actin and collagen α_1 _(I), respectively) differentially indicating functional heterogeneity [81]. Taken together, there is considerable uncertainty on the relation between HSC and MFB suggesting several distinct myofibroblast-like cell types. Their composition and functional role might be dependent on the nature of the underlying disorder [82].

### Contribution of bone marrow-derived cells to hepatic stellate cells, myofibroblasts, and fibroblasts in fibrotic liver tissue

Several studies have pointed to the bone marrow as a source of immature, multipotent cells in various organs. Bone marrow cells have the capacity to differentiate to hepatocytes, cholangiocytes, sinusoidal endothelial cells, and Kupffer cells, if the adequate micro-environment of the liver is present [83,84]. This phenomenon is of great importance for regenerative medicine (*e.g*., bone marrow stem cell therapy). It was recently extended for HSC and (myo-)fibroblasts under experimental and clinical conditions. By transplantation of genetically tagged bone marrow or of male bone marrow (Y-chromosome) to female mice, it was demonstrated that up to 30% of HSC in the liver originate from the bone marrow and acquire the MFB phenotype under injurious conditions [85]. Another study indicates that up to 68% of HSC and 70% of MFB in CCl_4_-cirrhotic mice liver derive from the bone marrow [86]. Even in human liver fibrosis a significant contribution of bone marrow cells to the population of MFB was proven, but it is presently unclear which type of specific bone marrow cells or mesenchymal stem cells is relevant for the generation of hepatic (myo-) fibroblasts [87]. Another experimental study shows that myelogenic fibrocytes are present in the liver, which can be differentiated by TGF-β to collagen-producing MFB [88]. They are a sub-population of circulating leucocytes, which display a unique surface phenotype with CD45^+ ^(haematopoietic origin), CD34^+ ^(progenitor cell), and type I collagen^+ ^(capability of matrix synthesis) [89], and exhibit potent immuno-stimulatory activities [90]. Fibrocytes represent a systemic source of contractile MFB in various fibrotic lesions, such as lung, keloids, scleroderma, and fibrotic changes of the kidney [91]. The mobilization of bone marrow cells and their recruitment into the damaged tissue is a general mechanism of tissue fibrosis and wound healing [92], which is most likely regulated by colony-stimulating factors (CSF), such as granulocyte-CSF (G-CSF) [93]. This mediator together with chemokines regulate the migration of bone marrow cells to sites of tissue injury, but also the efflux from the bone marrow into the circulation [90]. Activated HSC probably play an important role since these cells secrete a broad spectrum of inflammatory mediators (chemokines, M-CSF, SCF, PAF) and leukocyte adhesion molecules (ICAM-1, VCAM-1, NCAM) required for recruitment, activation, and maturation of blood-born cells at the site of injury [94]. The homing of myelogenic cells in the damaged liver was claimed to also have a positive effect on the resolution of liver fibrosis, since these cells express matrix metalloproteinases, which augment the degradation of fibrotic extracellular matrix [93].

### Contribution of peripheral blood cells to (myo-)fibroblasts of the liver

Recent studies indicate a highly developed multi-differentiation potential of a subgroup of circulating blood monocytes, which can be recruited quickly for tissue repair processes [95]. In addition, the content of circulating myelogenic stem cells in the blood is suggested to be important for regenerative mechanisms in consequence of ischemic and degenerative diseases (*i.e*., myocardial infarction). Investigations over the last years have proven that peripheral blood monocytes can be differentiated *in vitro *to hepatocyte-like cells if they are exposed with macrophage-colony stimulating factor (M-CSF) and specific interleukins (monocyte-derived neo-hepatocytes) [96,97]. Although for liver fibrogenesis not yet proven, subgroups of monocytes can differentiate into fibroblast-like cells (fibrocytes) after entering the damaged tissue. There they participate in fibrotic processes, *e.g*., of the lung and kidney. The differentiation is positively influenced by G-CSF, M-CSF, monocyte chemotactic peptide 1 (MCP-1), and other chemokines and haematopoietic growth and differentiation factors, which are expressed and secreted by activated HSC [28,98-100] and other liver cell types [101]. It is of interest that very recently an inhibitory effect of the acute-phase protein serum amyloid P (SAP) on the process of differentiation of monocytes to fibrocytes could be established [102] and, consequently, a preventive effect of SAP-injections on the development of bleomycin-induced lung fibrosis was found [103]. C-reactive protein (CRP) failed to show an inhibitory effect on the differentiation of monocytes to fibrocytes. Since SAP is synthesized in hepatocytes, severe liver injury might facilitate the monocyte-fibrocyte differentiation process due to reduction of the inhibitory SAP. Although this mechanism is presently somewhat speculative for the liver, circulating monocytes might nonetheless be a pool for immediate repair processes of liver damage. Beside special monocytes as source of fibroblasts in the fibrotic liver, circulating stem cells have to be considered, which are CD34^+ ^and CXCR4^+ ^(a chemokine receptor) [95]. G-CSF and the stromal derived factor (SDF)-1 are probably the most important regulators of stem cell mobilisation from bone-marrow and their integration into the damaged tissue followed by differentiation to fibroblasts and other cells.

### Epithelial-mesenchymal transition (EMT)

**Figure 7:**
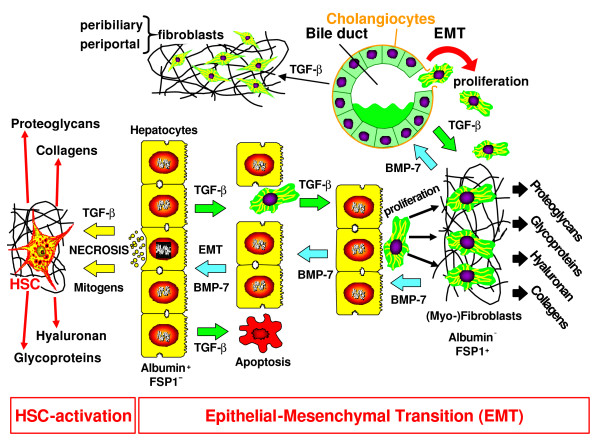
**Up-to-date mechanisms of fibrogenesis**. HSC activation, EMT, influx of fibrocytes, and differentiation of peripheral monocytes to fibroblasts at sites of injury. (Explanation is in the text).

Beside activation and transdifferentiation of HSC, a cell type, which is developmentally most likely derived from the *septum transversum *mesenchyme, from endoderm or from the mesothelial liver capsule [104], an increasing number of experimental studies points to an additional mechanism for the enlargement of the resident (local) pool of fibroblasts during the fibrotic reaction of the damaged organs, *e.g*., in kidney and lung [105]. This process, termed epithelial-mesenchymal transition (EMT), is well known in the context of embryonic development, but is now discussed as an important mechanism in the generation of fibroblasts during fibrogenesis in adult tissues [106] (Fig. 7). It was proven that in fibrotic kidney disease tubulus epithelial cells can transdifferentiate to fibroblasts expressing the fibroblast-specific protein 1 (FSP-1), also known as S100A4 calcium-binding protein, and are able to express collagens [106]. Similarly, alveolar epithelial cells of the lung are subject to EMT and also cardial endothelial cells can switch to fibroblasts under conditions of damage (mesenchymal-mesenchymal transition). It is estimated that in the kidney about 66% of fibroblasts are the result of EMT, in the heart the number climbs to about 20% (R. Kalluri, personal communication). *In *vitro and *in *vivo observations made in blood vessels following sustained inflammation support a hypothesis that endothelial cell transformation to myofibroblast-like cells may explain the increase of matrix proteins and of MFB pathognomonic of fibrotic diseases [107]. Very recent studies have also discussed EMT in liver fibrogenesis, after a transition of albumin-positive hepatocytes to FSP-1 positive and albumin-negative fibroblasts was shown. Preliminary studies claim that about 40% of hepatic fibroblasts derive from hepatocytes, but these data need further confirmation (R. Kalluri, personal communication). A very recent report provides evidence for EMT of mature mouse hepatocytes *in vitro *and of the mouse hepatocyte cell line AML12 [108]. The EMT-state was indicated by strong up-regulation of α_1_(I) collagen mRNA expression and type I collagen deposition. Thus, hepatocytes are capable of EMT changes and type I collagen synthesis. A further source of EMT are cholangiocytes (bile duct epithelial cells). In primary biliary cirrhosis (PBC) it was proven that bile duct epithelial cells express FSP-1 (S100A4) and vimentin as early markers of fibroblasts [109]. The bidirectional consequence of EMT of cholangiocytes are ductopenia (reduction of bile ducts) and enlargement of the pool of portal fibroblasts, which significantly contributes to portal fibrosis. *In vitro *studies with cultured human cholangiocytes have confirmed the clinical observations described. Thus, EMT proves to be a general pathogenetic principle of chronic cholestatic liver diseases [110]. In addition, activation and proliferation of portal/periportal mesenchymal cells to peribiliary MFB, which are stimulated in a paracrine manner by bile duct epithelial cells via TGF-β, PDGF-BB and endothelin-1 [111] turned out to be an important pathogenetic mechanism of portal fibrosis and septa formation in cholestatic liver diseases. Indeed, only a minority of ECM-producing MFB in obstructive cholestatic injuries are derived from HSC [112,113]. This also underlines the heterogeneous origin of MFB in fibrogenesis and emphasizes the importance of the underlying fibrogenic liver disease [82].

The molecular inducers of EMT are TGF-β [106], epidermal growth factor (EGF), insulin-like growth factor (IGF)-II, and fibroblast growth factor (FGF)-2, which promote the genetic and phenotypic programming of epithelial cells to mesenchymal cells (fibroblasts). The prototype of the most powerful inducer of EMT is TGF-β. The inducing function of TGF-β for the above described mesenchymal transition of mouse hepatocytes was shown by activation of Smad2/3 phosphorylation, inhibition by Smad4 silencing using siRNA and induction of the snail transcription factor [108]. Interestingly, TGF-β induces EMT only of those hepatocytes resisting to the pro-apoptotic effects of this cytokine [114,115]. The subpopulation of surviving hepatocytes exhibits an overexpression of Snail by TGF-β conferring resistance to programmed cell death [116]. Several additional pathways are involved in the generation of apoptosis resistance, *e.g*., proteinkinase A [114] and epidermal growth factor (EGF)/TGF-α [115]. Thus, EMT of hepatocytes is dependent on the balance between apoptotic and survival mechanisms. The process of EMT also requires the action of metalloproteinases and a TGF-β dependent down-regulation of E-cadherin both contributing to the release of epithelial cells from cell-cell and cell-basement membrane binding (Fig. 7). The most important molecular counterpart is the bone morphogenetic protein (BMP)-7, also belonging to the TGF-β superfamily. BMP-7 not only inhibits EMT, but can even induce a mesenchymal-epithelial transition (reverse EMT = MET) [117]. It has anti-apoptotic properties, anti-inflammatory and proliferation-stimulating effects [118]. BMP-7 inhibits TGF-β signalling via Smads [119], which transduce the effect of the latter cytokine from its receptor, a serine/threonine kinase, to the Smad-binding element (SBE) of respective target genes in the nucleus [120]. In addition, several trapping proteins such as the small proteoglycans decorin and biglycan, latency associated peptide (LAP), BAMBI (BMP and activin membrane-bound inhibitor), KCP (kielin-chordin-like protein), gremlin, and α_2_-macroglobulin change the balance between TGF-β and BMP-7 in favour of an anti-EMT effect due to binding a neutralization of TGF-β [121]. Similarly, the important downstream-modulator protein connective tissue growth factor (CTGF/CCN2) [122], which is expressed in hepatocytes, HSC, portal fibroblasts, and cholangiocytes [123,124] changes the functional TGF-β/BMP-7 ratio [125]. CTGF is over-expressed in experimental and human liver cirrhosis [126-128], which is mediated mainly by TGF-β, but also by endothelin-1, TNF-α, vascular endothelial growth factor (VEGF), nitrogen oxide (NO), prostaglandin E2, thrombin, high glucose, and hypoxia [129]. CTGF inhibits BMP, but activates TGF-β signalling by modulation of the receptor-binding of these ligands [123]. This is supported by very recent data, which show prominent antifibrotic effects of reduction of CTGF by siRNA [130,131]. Thus, depletion of CTGF greatly attenuates the development of experimental liver fibrosis. Taken together, both EMT, but also MET, in special conditions even MMT (mesenchymal-mesenchymal transition, *e.g*., vascular endothelial cells to fibroblasts), and the fine tuning of the bioactive TGF-β/BMP-7 ratio and of their adaptor- and trapping proteins offer multiple regulatory possibilities of influencing fibrogenesis. These mechanisms are known in some detail for the kidney [132], but need more experimental proof for the liver, in particular with regard to its quantitative contribution to fibrogenesis.

### Options for diagnostic and therapy

Newly recognized pathogenetic mechanisms of fibrosis described above provide several innovative options for therapy of liver fibrogenesis and non-invasive diagnostic strategies (Table 1). The determination of the TGF-β/BMP-7 ratio in serum or plasma is potentially promising, since this ratio might reflect the process of EMT and, thus, at least partially the rate of progression of fibrosis. A decrease of this ratio might indicate those patients with slow progression (slow fibroser), an increase a fast progression (rapid fibroser). However, some precautions have to be considered. The cytokine ratio in the circulation might be not an accurate reflection of their activity at the immediate environment of epithelial cells and fibroblasts, respectively, and major fractions of these cytokines might be in a biologically latent form. Thus, the protein ratio does not necessarily mimic the diagnostically important activity ratio of these mediators.

**Table 1:** Therapeutic and diagnostic options based on newly identified pathogenetic mechanisms of liver fibrosis

**Parameter**	**Pathobiochemical basis**	**Potential serum markers of fibrosis**	**Therapeutic approach**
TGF-β	Fibrogenic master cytokine, up-regulation in fibrotic liver; inducer of epithelial-mesenchymal transition (EMT)	Elevation by up-regulation in the fibrotic liver, release from necrotic hepatocytes and reduced hepatic clearance	Inhibition of TGF-β, blockade of intracellular signalling
BMP-7	TGF-β antagonist: anti-apoptotic; anti-inflammatory; anti-EMT	Elevation in serum, indicator of slow fibrosis?	BMP-7 or BMP-7 peptide fragments antagonize TGF-β, antifibrotic effect, stimulation of liver regeneration
TGF-β/BMP-7 Ratio	Determines epithelial-mesenchymal transition (EMT) and profibrogenic action of TGF-β	Potentially of prognostic significance for estimation of the progression rate of fibrosis (rapid versus slow fibrosis)	Modulation of the ratio by addition of recombinant BMP-7 has an antifibrotic effect
CTGF	Down-stream modulator protein of TGF-β, influences functional TGF-β/BMP-7 ratio by elevation of TGF-β and decrease of BMP-7 action	Elevation under conditions of active fibrogenesis, decrease with advancing cirrhosis and in the terminal stage without fibrogenic activity	Inhibition of CTGF expression by siRNAs or blocking with humanized monoclonal anti-CTGF antibodies (FG-3019, FibroGen); has a strong antifibrotic effect
Fibrocytes	Bone marrow-derived progenitor cells of fibroblasts increase the pool of fibroblasts in the fibrotic liver	Flow-cytometric detection of CD34^+^, CD45^+^, and collagen-1^+ ^cells in peripheral blood or buffy coat leucocytes; potential indicator of increased influx into the damaged liver tissue	Hormonal modulation of release of fibrocytes from bone marrow and integration into the liver?
G-CSF	Recruitment of bone marrow-derived cells in the circulation and stimulation of their homing in the fibrotic liver tissue	Elevated concentrations, relation to fibrogenesis not yet established	G-CSF triggered haematopoietic stem cells or G-CSF itself accelerates healing of experimental liver damage and improves the survival rate

The determination of CTGF in serum or plasma is suggested as a further innovative parameter of fibrogenesis, since this modulator protein is strongly up-regulated in the fibrotic liver, synthesized and secreted by parenchymal and non-parenchymal cells [124] and since the action of the profibrogenic TGF-β is stimulated but that of the antifibrogenic BMP-7 is inhibited [123]. Preliminary studies point to significantly enhanced concentrations of CTGF in blood of patients with active liver fibrogenesis [133] in contrast to advanced cirrhosis with low activity of active fibrogenesis, which is reflected by a relative decrease of serum CTGF.

The flowcytometric detection of circulating fibrocytes in blood or in buffy coat leucocytes by using CD34^+^, CD45^+^, and collagen I positivities as identifying markers might be a way for evaluation of their diagnostic potential. Alternatively, these antigens might be detected by amplifying their mRNA using a quantitative PCR approach. In addition, a re-evaluation of the high concentrations of G-CSF, GM-CSF, and M-CSF in serum of cirrhotic patients published previously [134] as mobilizers of bone marrow cells and fibrocytes and of their integration into the damaged liver tissue [135] might be a promising task. It should be analyzed whether a systemic elevation of the haematopoietic growth factors correlates with the activity of liver fibrogenesis.

Numerous publications discuss anti-fibrotic therapeutic strategies by inhibition of TGF-β [9,67,136-138], but the systemic application of inhibitors and consequently an overall and ubiquitous reduction of TGF-β activity will most likely have severe side effects, *i.e*., on tumor development and progression, auto-immunopathy and degenerative diseases [139]. Therefore, the therapeutic application of recombinant human BMP-7 or functionally active BMP-7 fragments might be advantageous since BMP-7 inhibits experimental fibrosis in rats [140], stimulates liver regeneration [118], and inhibits TGF-β-driven parenchymal cell apoptosis due to its antagonism of TGF-β. Experimental trials with thioacetamide-induced rat liver fibrosis point to successful antifibrotic results [140]. Similarly, extensive studies with experimental kidney diseases prove that BMP-7 can induce MET and, thus, has regenerative and antifibrotic effects [141]. Presently, it is not known whether the positive CTGF-inhibitory experiments for suppression of experimental fibrosis [130,131] can be translated into clinical practice, but studies – in which CTGF activity is reduced by systemic application of a humanized, monoclonal, blocking antibody (F-3019), which neutralizes and accelerates the clearance of this protein [142] – are in progress and point to successful preliminary results. Pathophysiologically, the inhibitors of CTGF should have fibro-suppressive effects since the TGF-β/BMP-7 ratio is switched in favour of BMP-7. This was recently shown by inhibition of CTGF expression [130,131]. In conclusion, further intensive studies are required to translate the positive results of cell culture studies and of animal experiments into clinical application. The new pathogenetic insights justify strong optimism since the spectrum of potential approaches to interfere with the fibrogenic pathway are greatly broadened.

## Conclusion

The above described changing view on the pathogenetic mechanisms of liver fibrosis clearly suggests that one has to reconsider the exclusive role of HSC in the development of fibrosis. Although some of the newly proposed fibrogenic mechanisms have to be consolidated by additional experimental evidence in vitro and in situ, they indicate the presence of distinct subpopulations of myofibroblasts/fibroblasts in fibrosing liver, of which HSC-derived fibrogenic cells are only one of several sources. Most important, the composition of (myo-)fibroblasts may vary with the etiology of fibrosis, *e.g*., primary biliary cirrhosis might activate a pathogenetic pathway different from alcoholic fibrosis. These facts point to the important notion that results obtained with various models of experimental fibrogenesis cannot be generalized because different classes of (myo-)fibroblasts are generated by diverse pathways. Furthermore, HSC-activation in culture cannot be regarded any longer as the almost dogmatic paradigm of the liver fibrogenic mechanism as it was in the past. Since now detailed information on the molecular cascades of intracellular fibrogenic signaling is available, we have learned that several of them are modulated cell-type specifically. Therefore, it is conceivable that distinct subpopulations of fibroblasts and their transient precursor cell types respond differently to major fibrogenic cytokines, *e.g*., TGF-β. If this is the case, the complexity of the fibrogenic mechanisms will increase strongly in the future and the experimental conditions have to be described in detail. Taken together, studies on fibrogenesis in the liver (and other organs as well) are now pushed forward a lot, hopefully resulting in new impulses for therapy and diagnosis.

## Competing interests

The author(s) declare that they have no competing interests.

## Authors' contributions

All the authors contributed equally to this work. All authors read and approved the final manuscript.
